# The effect of Rift Valley fever virus Clone 13 vaccine on semen quality in rams

**DOI:** 10.4102/ojvr.v82i1.919

**Published:** 2015-06-15

**Authors:** Geoff Brown, Estelle H. Venter, Paul Morley, Henry Annandale

**Affiliations:** 1 Department of Production Animal Studies, University of Pretoria, South Africa; 2 Department of Veterinary Tropical Diseases, University of Pretoria, South Africa; 3 Diagnostic Medicine Center, Colorado State University, United States; 4 Onderstepoort Veterinary Academic Hospital, University of Pretoria, South Africa

## Abstract

Rift Valley fever (RVF) is an arthropod-borne viral disease of importance in livestock and humans. Epidemics occur periodically in domestic ruminants. People in contact with infected livestock may develop disease that varies from mild flu-like symptoms to fatal viraemia. Livestock vaccination may assist in disease control. Rift Valley fever virus (RVFV) Clone 13 is a relatively new vaccine against RVF, derived from an avirulent natural mutant strain of RVFV, and has been shown to confer protective immunity against experimental infection with RVFV. The hypothesis tested in the current trial was that rams vaccinated with RVFV Clone 13 vaccine would not experience a reduction in semen quality (measured by evaluating the percentage progressively motile and percentage morphologically normal spermatozoa in successive ejaculates) relative to unvaccinated control animals. Ram lambs were screened for antibodies to RVFV using a serum neutralisation test. Animals without detectable antibodies (*n* = 23) were randomly allocated to either a test group (*n* = 12) or a control group (*n* = 11). Animals in the test group were vaccinated with RVFV Clone 13 vaccine. Daily rectal temperature measurements and weekly semen and blood samples were taken from all animals. Seven animals were eliminated from the statistical analysis because of potential confounding factors. Logistic regression analysis was performed on data gathered from the remaining animals to determine whether an association existed between animal group, rectal temperature and semen quality parameters. No correlation existed between the treatment group and values obtained for the semen quality parameters measured. There was no statistically significant post-vaccination decline in the percentage of live morphologically normal spermatozoa, or the percentage of progressively motile spermatozoa, either when assessed amongst all animals or when assessed within individual groups. A repeat study with a larger sample size and a more comprehensive pre-screening process may be indicated to avoid the inclusion of unsuitable animals.

## Introduction

Rift Valley fever (RVF) (*Phlebovirus*, *Bunyaviridae*) is a mosquito-borne viral disease of ruminants with significant economic and public health implications. The virus may cause fatal disease in juvenile and adult animals and may induce abortion in pregnant animals (Flick & Bouloy 2005). It is also a zoonosis that may have debilitating or life-threatening effects (Gerdes 2004). People who live and work with livestock are at greatest risk of contracting the disease (Swanepoel & Coetzer 2004).

Vaccination of livestock helps prevent the spread of disease by reducing the population of viraemic animals that may infect vectors. Vaccination of dams confers colostral immunity to offspring, which reduces juvenile mortality (Gerdes 2004). In South Africa, three vaccines are available: a modified-live vaccine (MLV), a killed vaccine, and a vaccine derived from an avirulent natural mutant strain of Rift Valley fever virus (RVFV Clone 13).

RVFV Clone 13 vaccine (Onderstepoort Biological Products, Onderstepoort, South Africa) is produced from the avirulent strain 74HB59 of RVFV, derived originally by passage of a non-fatal human case of RVF in the Central African Republic through mice and Vero cells. This strain lacks approximately 70% of the S-segment of the ribonucleic acid (RNA) genome of the virus coding for the non-structural protein NSs, which was found to be the determinant of virulence (Ikegami & Makino 2009; Von Teichman *et al.* 2010). This deletion renders the virus unable to revert to virulence *in vivo* (Pepin *et al.* 2010).

Several bacterial, viral and protozoal diseases have been shown to cause a reduction in semen quality in mammals. This may be mediated by a local effect on scrotal thermoregulation, as in bovine scrotal cutaneous *Dermatophilus congolensis* infection (Sekoni 1993) or the hypothesised effect of scrotal oedema caused by *Eperythrozoon wenyonii* infection (Montes *et al.* 1994). Ovine orchitis caused by *Arcanobacterium pyogenes* was shown to cause a reduction in semen quality, either directly through bacterial invasion of spermatogenic tissue or by general inflammation caused by epididymo-orchitis (Gouletsou *et al.* 2004). Arteriviruses, such as equine viral arteritis or porcine respiratory and reproductive syndrome, have a tropism for testicular tissue that may directly affect spermatogenesis (Prieto & Castro 2005). Bluetongue disease has been associated with infertility in male ruminants. This may occur either as a result of systemic pyrexia or microvascular lesions in the testis caused by orbivirus infection (Kirschvink, Raes & Saegermann 2009; Osburn 1994).

Vaccination with MLV vaccines has been cited by anecdotal accounts and proven by controlled trials to cause alterations in the spermiogram. Vaccination with a MLV strain of bluetongue virus was shown to cause a significant transient reduction in semen quality (Bréard *et al.* 2007). By contrast, the use of an inactivated bluetongue vaccine was reported to have no detrimental effect on semen quality (Leemans *et al.* 2012). Interestingly, vaccination of boars with a live, recombinant pseudorabies virus vaccine resulted in no significant difference in semen quality pre-vaccination and post-vaccination (Castro *et al.* 1992). The use of inactivated porcine circovirus vaccines was found by researchers to have no statistically significant effect on semen quality (Caspari *et al.* 2014).

The purpose of the current study was to determine the effects (if any) of RVFV Clone 13 vaccine on semen quality in rams, and to quantify such effects by measuring differences in semen motility, sperm morphology and rectal temperature over a period of approximately one ovine spermatogenic cycle of 42 days.

## Materials and methods

### Experimental animals

Merino ram lambs aged between 9 and 14 months from a reserve flock were tested for antibodies to RVFV using a serum neutralisation test (SNT). Twenty-eight animals were tested once using the SNT, and seropositive animals were excluded from participation in the study.

Animals were not screened for antibodies to any other diseases prior to inclusion in the trial.

The resultant 23 seronegative rams were randomly divided into two groups of approximately equal size:

- Group 1: Animals vaccinated with RVFV Clone 13 vaccine (*n* = 12).
- Group 2: Unvaccinated control animals (*n* = 11).

Semen was collected from all animals prior to vaccination of test animals. Semen was then collected at weekly intervals for 42 days after vaccination.

## Ethical consideration

Ethical approval for the study was obtained from the University of Pretoria Research Ethics Committee (Project V020-12). All procedures were carried out by a veterinarian. The study was performed at Knoffelfontein Farm, Philipstown, Northern Cape, South Africa. This facility is a Department of Agriculture, Forestry and Fisheries approved centre for assisted animal reproduction. All animals were housed together in an outdoor pen with sufficient shelter and *ad libitum* access to food and water.

### Vaccination procedure

On day 0 of the study, animals in the test group (*n* = 12) were vaccinated by subcutaneous injection with RVFV Clone 13 vaccine (Onderstepoort Biological Products) (OBP) (batch number not specified). Vaccine administration was performed as directed by the manufacturer, using a 3 mL syringe (Braun Omnifix^®^, Melsungen, Germany) and a three quarters inch 21 gauge needle (Terumo, Louvain, Belgium). Animals in the control group (*n* = 11) were unvaccinated. No inert placebo was used.

### Blood collection

Blood was collected weekly by jugular venipuncture. Approximately 3 mL of blood per ram was aspirated into a plain serum tube (Vacutainer, Becton-Dickinson, Woodmead, South Africa). Tubes were refrigerated at 4 °C for 48 hours until separation of serum and cellular components occurred. Serum was placed in a fresh tube and frozen at -18 °C until delivery to the laboratory.

### Antibody testing

Antibody testing was performed using a SNT at the Virology Laboratory of the Department of Veterinary Tropical Diseases (DVTD), University of Pretoria, according to the OIE Terrestrial Manual (World Organisation for Animal Health [OIE] 2013) and slightly modified to the standard operating procedures (SOP) of the laboratory.

### Semen collection

Semen was collected by means of an artificial vagina (AV) or by electrostimulation. Where an AV was used, one ram at a time was introduced into a pen containing a restrained ewe in oestrus. Some rams showed normal sexual behaviour and attempted to mount the ewe, whereupon the penis was diverted into the AV and semen was collected in a warmed collection vial.

In animals where AV semen collection failed, a lubricated electroejaculation probe (Ruakara Ram Probe^®^, Shoof International, Cambridge, New Zealand) was introduced into the rectum to the level of the prostate. Electrical stimulation was intermittently applied until ejaculation occurred. Semen was collected from an exteriorised penis into a warmed collection vial.

### Semen evaluation

Semen evaluation comprised assessment of individual progressive motility and sperm morphology. All evaluations were performed by the same operator in order to ensure repeatability and consistency.

All equipment used in semen handling was warmed to 37 °C. Findings were recorded on a semen evaluation form according to the format set out by Nöthling and Irons (2008) and entered into a computer spreadsheet (Excel^®^ 2010, Microsoft Corporation, Redmond, Washington).

Individual sperm motility was evaluated by examining a droplet of extended semen (Triladyl^®^, Minitüb, Tiefenbach, Germany). A single droplet of extended semen was placed between a coverslip and microscope slide. Ten fields at 200× magnification phase-contrast microscopy were evaluated according to a method previously described (Nöthling & Dos Santos 2012). The percentages of individual progressive, aberrant and immotile sperm per field were estimated and recorded. The mean values for 10 fields described the subjective net motility of the ejaculate.

Sperm morphology was evaluated using 1000× oil-immersion bright-field light microscopy of an eosin-nigrosin stained smear, fixed under a coverslip using a mounting medium (Entellan^®^ Merck Millipore International, Billerica, Massachusetts).

Two hundred spermatozoa were evaluated per ejaculate. Percentages of morphologically normal sperm, live morphologically normal sperm, sperm with nuclear defects, and sperm with tail or acrosomal defects were calculated.

### Exclusion of animals from statistical analysis

Animals with progressive motility of less than 50% on day 0 were excluded from statistical analysis. Such animals would not pass a breeding soundness examination under real-world conditions, and motility was deemed unlikely to improve significantly with repeated collections.

Animals that showed antibodies to RVFV prior to the onset of the trial (but after the initial screening process) were excluded from statistical analysis.

Animals that displayed a fever response for five or more consecutive daily measurements, starting at day 0, were excluded in order to remove persistent fever as a confounding factor. A reference of 39.1 °C ± 0.5 °C was used for normal ovine rectal temperature; any temperature greater than or equal to 39.6 °C was considered a fever response (Kahn & Line 2005).

### Statistical procedures

Data were analysed using Statistical Analysis Software (SAS) (SAS Institute, Cary, North Carolina). Descriptive statistics were generated from the data using SAS plugin for MS Excel. The effect of vaccination on percentage progressively motile spermatozoa was assessed using repeated-measures logistic regression analysis (SAS GENMOD function).

The primary outcome assessed was whether vaccination affected breeding soundness. This could not be directly assessed, as the intervening variable of temperature existed, and therefore it was assessed whether animals with higher body temperatures also had poorer progressive semen motility.

A secondary outcome assessed was whether vaccination had any effect on temperature. From this, a conclusion could be drawn on whether vaccinated animals had poor semen quality relative to unvaccinated animals.

## Results

### Clinical findings

Animals used in the study were young, peripubertal ram lambs. Only three out of the 23 animals that passed the initial antibody screening process were recorded as having two permanent teeth; the remainder had only deciduous teeth. The modal body condition score for the entire group of rams was 2.5 out of 5. The mean scrotal circumference of all animals was 29.1 cm.

No swelling, pain or redness was noted at vaccination sites in any vaccinated animals at any time during the study period. No animal became sufficiently ill at any time during the trial to warrant treatment or exclusion from the trial.

### Temperature variation

The mean rectal temperature of all animals (in both the vaccinated and the control group) throughout the study period was 39.34 °C ± 0.41 °C (mean ± s.d.). A maximum of 41.4 °C was measured in one animal on day 2 of the trial period, and a minimum of 37.4 °C measured in one animal on day 24 of the trial period.

The mean daily rectal temperature of all animals in the control group throughout the study period was 39.27 °C ± 0.36 °C (mean ± s.d.). The mean daily rectal temperature of all animals in the vaccinated group throughout the study period was 39.41 °C ± 0.43 °C (mean ± s.d.). The highest daily average rectal temperature recorded in all animals (in both the vaccinated and the control group) throughout the study was a temperature of 39.73 °C ± 0.35 °C (mean ± s.d.), recorded on day 29.

### Antibody testing

One animal from the control group tested positive using SNT for RVFV antibodies, without exposure to vaccine antigen. Of the vaccinated animals, all except two exhibited an antibody response. Most vaccinated animals displayed a measurable antibody titre on the third test (day 21 after vaccination) (Table 1).

**TABLE 1:** Summary of serum neutralisation test results.

Type	Ram ID	Day 0	Day 6	Day 14	Day 21	Day 28	Day 34	Day 43
Control animals	B016	Neg	Neg	Neg	Neg	Neg	Neg	†
	B049	Neg	Neg	-	Neg	Neg	Neg	Neg
	B054	Neg	Neg	-	Neg	Neg	Neg	Neg
	B057	Neg	Neg	Neg	Neg	Neg	Neg	Neg
	B070	Neg	Neg	Neg	Neg	Neg	Neg	Neg
	B100	Neg	Neg	Neg	Neg	Neg	Neg	Neg
	B205	01:05	01:10	-	01:14	01:10	01:14	Neg
	B208	Neg	Neg	Neg	Neg	Neg	Neg	Neg
	B214	Neg	Neg	Neg	Neg	Neg	Neg	Neg
	B260	Neg	Neg	-	Neg	Neg	Neg	Neg
	B341	Neg	Neg	Neg	Neg	Neg	Neg	-
Vaccinated animals	B043	Neg	Neg	01:40	01:28	01:14	01:28	01:20
	B104	Neg	Neg	01:20	01:14	01:20	01:20	01:20
	B122	Neg	Neg	-	01:28	0.09722222	0.11944444	0.09722222
	B134	Neg	Neg	01:10	01:07	01:07	01:14	Neg
	B253	Neg	Neg	01:20	01:20	01:28	01:14	01:10
	B259	Neg	Neg	01:40	01:40	01:56	01:28	01:40
	C002	Neg	Neg	-	0.11944444	0.09722222	01:56	01:28
	C003	Neg	Neg	Neg	Neg	Neg	Neg	Neg
	C006	Neg	Neg	01:28	01:20	01:10	01:10	Neg
	C009	Neg	Neg	Neg	Neg	Neg	Neg	Neg
	C013	Neg	Neg	01:14	01:20	01:28	01:20	01:40
	C014	Neg	Neg	Neg	01:14	01:14	01:07	01:07

†, No value indicates that sample was of inadequate quality for testing.

### Semen collection

Electroejaculation (EE) was the predominant means of semen collection throughout the study. Of the 161 semen collections performed during the study, 115 were by EE and 46 were by AV.

### Exclusions from statistical analysis

One ram from the control group was excluded from the trial because it seroconverted between the screening process and the first blood sampling. Five animals were excluded because they had poor semen quality (< 50% progressively motile) on day 0 of the trial. One animal was excluded because of a persistent febrile response noted from day 0 of the trial.

### Descriptive statistics: Semen evaluation data

Descriptive statistics were generated for both vaccinated and control groups, grouped together. The descriptive statistics for progressive motility and live morphologically normal sperm are displayed in Table 2.

There was no significant temporal decline in the mean number of live, morphologically normal spermatozoa and the mean number of progressively motile spermatozoa throughout the trial (Tables 3 and 4).

**TABLE 2:** Percentage progressive motility and live, morphologically normal sperm of vaccinated and control groups assessed together.

Parameter	Day 0 value	Cumulative values across entire study
Mean progressive motility	63.94	68.6
s.d.: progressive motility	11.26	19.98
Mean morphologically normal	70.05	68.76
s.d.: morphologically normal	14.76	14.38

s.d., standard deviation.

**TABLE 3:** Percentage progressively motile spermatozoa.

Type	Ram ID	Day 0	Day 6	Day 14	Day 21	Day 28	Day 34	Day 43	Average
Control animals	B049	75.5	72.5	68	30	78.5	83	81.5	69.86
	B054	64	76.5	64.5	64	84.5	84	83	74.36
	B057	58.5	83.5	63.5	81	90	70	84	75.79
	B070	67.5	61.5	74.5	76.5	86.5	76	69.5	73.14
	B100	51.5	84.5	81	9.6	84	63	80.5	64.87
	B208	60	78	31.5	22	20	72	65	49.79
	B214	69.5	78	81	81	78	80.5	68	76.57
	B260	56.5	34.5	35	34.5	47	60	76	49.07
	B341	54	17.5	10.5	66	40	59.5	54	43.07
Vaccinated animals	B043	82	76	81	82	74	80	68	77.57
	B104	81.5	72.5	74	48.5	79	83	80	74.07
	B122	58	89	62	72.5	86	85	88	77.21
	B134	63.5	79	69.5	28.5	85	70	29.5	60.71
	B253	59	70	70	39.5	75	81.5	84	68.43
	B259	74	64	80	77.5	75	78.5	76.5	75.07
	C009	50.5	21	59	27	37.5	30	3.5	32.64

**TABLE 4:** Percentage morphologically normal sperm throughout trial.

Type	Ram ID	Day 0	Day 6	Day 14	Day 21	Day 28	Day 34	Day 43	Average
Control animals	B049	93	92	92.5	93.5	97.5	98.5	96.5	94.79
	B054	96.5	98	98.5	91	98.5	94.5	99	96.57
	B057	97.5	98.5	92.5	89	95	96	98.5	95.29
	B070	98	97	93.5	94	97.5	97	98.5	96.5
	B100	97.5	99	99.5	99	96.5	97	100	98.36
	B208	96.5	98	94	95	75	95.5	94.5	92.64
	B214	85	93.5	98.5	91	80.5	87.5	81	88.14
	B260	93.5	97.5	99.5	99	94.5	96.5	99	97.07
	B341	41.5	44.5	38	87.5	62.5	81	68.5	60.5
Vaccinated animals	B043	98	98	97	99.5	94.5	98	95	97.14
	B104	98	98.5	98.5	97	92.5	96	99	97.07
	B122	99	97.5	90	89.5	95.5	98.5	96.5	95.21
	B134	96	97.5	94.5	97	97	96.5	97.5	96.57
	B253	78.5	90	82	76.5	89.5	96.5	98.5	87.36
	B259	89	89	98	92	96.5	96	98	94.07
	C009	89.5	87	62.5	66.5	68.5	62	61	71

### Logistic regression analysis

When animals (both vaccinated and control) were grouped together and evaluated as a single group, progressive sperm motility on day 0 of all animals was found to correlate strongly with progressive motility after day 0 throughout the trial (*p* = 0.0062).

When progressive motility throughout the trial was evaluated according to group (vaccinated vs control), no significant difference was found between groups (*p* = 0.0499), therefore animal group was not shown to correlate with a difference in percentage progressively motile spermatozoa.

When compared within groups, values for progressively motile on day 0 were shown to correspond significantly with subsequent values through the trial (*p* = 0.0321).

Daily temperature values were not found to have a significant association with group (*p* = 0.8606).

The occurrence of fever (rectal temperature ≥ 39.6 °C) on any day throughout the trial was not found to have a significant association with animal groups (vaccinated or control) (*p* = 0.6665).

When treatment group as well as progressive motility on day 0 were evaluated in the same multivariable regression model for their effects on progressive motility throughout the trial, it was found that treatment group was not significantly associated with a reduction in progressive motility (*p* = 0.3325), but that progressive motility on day 0 correlated significantly with subsequent progressive motility (*p* = 0.0321) (Table 5).

**TABLE 5:** Summary of correlations between measured variables: single variable models.

Variable 1	Variable 2	*p*-value
Day 0 progressive motility	Post-day 0 progressive motility	0.0062
Post-day 0 progressive motility	Treatment group	0.0499
Within-group day 0 progressively motile	Within-group post-day 0 progressively motile	0.0321
Daily temperature value	Treatment group	0.8606
Fever occurrence	Treatment group	0.6665

Body temperature (measured once a week, on the day of semen collection) and the values for progressively motile sperm on day 0 were evaluated in a two-variable regression model to assess their combined effect on the percentage of progressively motile sperm through the study. In this model it was found that the weekly measured temperature was not significantly correlated with progressive motility throughout the trial (*p* = 0.3711), but that the values for progressively motile sperm on day 0 from a given animal correlated strongly with subsequent pro­gressive motility from that animal evaluated during the trial (*p* = 0.0002).

Similarly, body temperature (measured once a week, on the day of semen collection) and the values for progressively motile sperm on day 0 were evaluated in a two-variable regression model for their combined effect on percentage live, morphologically normal sperm throughout the trial. It was found that the weekly temperature value was not significantly correlated with the value for live, morphologically normal sperm through the study (*p* = 0.8785) but that progressive motility on day 0 correlated significantly with the value for live, morphologically normal sperm throughout the study (*p* = 0.0190).

## Discussion

Very few research trials have been performed involving RVFV Clone 13 vaccine (Dungu *et al.* 2010; Von Teichman *et al.* 2011). The current trial did not evaluate the protective effect of RVFV Clone 13 vaccine by exposing animals to a challenge trial.

Obtaining enough animals proved difficult, as they needed to be unvaccinated, serologically naïve rams. Some rams were therefore too young to reliably pass a breeding soundness examination. This complicated the data analysis, as a significant number of animals needed to be excluded from statistical calculations on the grounds of poor semen quality at the start of the trial.

A large proportion of younger animals were randomly allocated to the vaccinated group. This resulted in more exclusions for poor semen quality occurring in this group. An improvement on the implemented randomisation model may have been to allocate animals to two groups based on the results of their initial semen quality analysis, to ensure an even distribution between groups prior to the administration of the vaccine to one of the groups.

Various factors may have caused poor semen quality in a large proportion of trial animals. Male animals store sperm in the epididymides prior to ejaculation. Prolonged sperm storage (for example lack of ejaculation as a result of absent or reduced sexual activity) may result in ‘aged’ spermatozoa that exhibit a lower than normal progressive motility and a higher proportion of epididymal defects and loose heads. This phenomenon was reviewed by Barth and Oko (1989), and an evolutionary biology review characterised such defects as ‘post-meiotic senescence’ (Pizzari *et al.* 2007). Hence, repeated semen collections may have resulted in an improvement in semen quality as aged spermatozoa were ejaculated and replaced with newly produced spermatozoa.

Importantly, younger animals with small scrotal circum­ferences and no permanent teeth were over-represented amongst those excluded for poor semen quality. This might suggest that these animals were not adequately mature prior to first semen collection and evaluation.

In addition to the physiological reasons for poor semen quality discussed above, events during collection and handling of the semen sample may affect its quality. Animals that have their semen collected by EE are more likely to contaminate the sample with urine or produce an oligospermic ejaculate that consists mainly of accessory gland fluid. An AV is well known to provide a more consistent semen sample than EE, which is more reflective of true semen quality (Hulet, Foote & Blackwell 1964). Had a larger pool of animals been available, a better option may have been to exclude animals that failed to mount an AV, and then to randomise animals by semen quality as discussed above.

A single animal from the control group was excluded from the statistical analysis because of seroconversion to RVFV. This suggests that the animal was exposed to RVFV between the initial pre-enrolment screening test and the first pre-vaccination test. Assuming high sensitivity and specificity of the SNT, the fact that no other animals exhibited antibody titres at this stage of the trial might indicate a low-intensity occurrence of RVFV in the region where the trial was conducted. Alternatively, an imperfect sensitivity of the SNT test used may have resulted in a false-negative result at the initial screening, which could have been avoided by multiple testing or the parallel use of another diagnostic test.

When temperatures were averaged amongst groups and all animals were included (including those excluded from subsequent statistical analysis), it was found that throughout the trial, vaccinated animals had a slightly higher average temperature (39.41 °C ± 0.43 °C) than control animals (39.27 °C ± 0.36 °C). The significance and underlying causes of this finding are open to debate. The authors speculate that this is purely as a result of natural variation and would normalise with increased sample size; however, some vaccine effect cannot be ruled out.

Importantly, when logistic regression analysis was performed, it was found that rectal temperature had no correlation with either progressive motility or percentage live, morphologically normal sperm. This suggests that a febrile response to vaccination (if any) was minimal and was insufficient to induce abnormalities in the spermiogram.

Interestingly, in prior work by Dungu *et al.* (2010), no Clone 13 vaccinated animals exhibited temperature reactions above 40 °C at any stage of the trial. There were several instances in the present study in which rectal temperatures in both control and vaccinated animals were elevated above 40 °C. This may be as a result of significant differences in environmental conditions under which animals were kept. In the trial by Dungu *et al.* (2010), animals were kept in an indoor, temperature-controlled confinement facility as a virulent infective virus was used. In the present trial, animals were kept outdoors during the early Karoo summer, and had to contend with significant variation in environmental temperature.

As discussed previously, Leemans *et al.* (2012) found that inactivated bluetongue virus had no adverse effect on semen quality in rams. The authors attributed this outcome to an absence of febrile response to vaccination, compared to the findings of a previous study using live vaccine by Bréard *et al.* (2007) and the effect of a natural infection (Kirschvink *et al.* 2009).

This result contrasts with the findings of Dungu *et al.* (2010) and Von Teichman *et al.* (2011) that sheep and calves vaccinated with the live (Smithburn) RVFV vaccine failed to develop a post-vaccination temperature response. No previous research has been performed that evaluates the effect of a live RVFV vaccine on semen quality, but it is known that the live RVFV vaccine may induce teratogenesis in pregnant animals, and its use should be avoided in these animals if at all possible (Coetzer & Barnard 1977).

It may therefore be hypothesised that, as the live RVFV vaccine was not found to induce a febrile response in sheep or calves, it may be safe for use in male animals used for breeding. This line of reasoning may require further investigation.

The day 0 value for percentage progressive motility in this study was found to be highly predictive of subsequent values for progressive motility throughout the trial (*p* = 0.0062) when both groups (vaccinated and control) were assessed together. Similarly, the day 0 value for percentage progressively motile sperm was found to be predictive of subsequent values for this parameter (*p* = 0.0321) when assessed within the group. These findings suggested that an animal with high-quality semen on day 0 was likely to continue to produce high-quality semen throughout the trial, and an animal with poor-quality semen on day 0 was likely to continue to produce poor-quality semen. Interestingly, there was a right-shift in distribution of percentage progressive motility when comparing values on day 0 and values after day 0 amongst both control and vaccinated groups, indicating a temporal improvement in semen quality amongst all animals as the trial progressed. As discussed previously, this apparent improvement may have occurred as a result of repeated ejaculations, thereby eliminating aged epididymal sperm that would have shown poor progressive motility.

Two animals from the vaccinated group failed to display measurable antibody titres to RVFV at any stage during the trial. These animals were subsequently excluded from the trial on the grounds of poor semen quality. However, the fact that these animals failed to seroconvert after vaccination is worthy of special mention.

In previously reported work (Dungu *et al.* 2010), two out of 17 vaccinated animals exhibited a weak antibody response to a 10^6^ PFU dose of RVFV Clone 13 vaccine, which was nonetheless able to protect them against challenge with live virus. In an earlier study by Barnard (1979), it was noted that after two inoculations with the Smithburn live virus vaccine, two out of five cattle failed to develop an antibody response detectable by the SNT. These animals were nonetheless immune when challenged with live virus. One reason for this might be that the SNT is not sensitive enough in detecting neutralising antibodies. Alternatively, antibody-mediated humoral immunity may not be the most important immune response that protects animals against RVFV infection.

## Conclusion

Clone 13 vaccine was found to be capable of inducing seroconversion in vaccinated rams. These animals did not experience significant deterioration in semen quality post-vaccination. Therefore, according to these findings, RVFV Clone 13 is a vaccine that can be used safely in breeding rams.

Conclusions drawn from this trial must be interpreted in the context of its small sample size.

The fact that two animals out of 12 failed to seroconvert within 42 days after vaccination warrants further investigation. A challenge trial may assist in confirming whether or not the vaccine is protective. A repeat of the trial with a larger sample size may confirm, with greater statistical certainty, that the vaccine has no ill effect on semen quality parameters while remaining effective in protecting animals against clinical disease.
